# Whale Optimization for Cloud–Edge-Offloading Decision-Making for Smart Grid Services

**DOI:** 10.3390/biomimetics9050302

**Published:** 2024-05-18

**Authors:** Gabriel Ioan Arcas, Tudor Cioara, Ionut Anghel

**Affiliations:** 1 Bosch Engineering Center, 400158 Cluj-Napoca, Romania; gabriel.arcas@ro.bosch.com; 2 Computer Science Department, Technical University of Cluj-Napoca, Memorandumului 28, 400114 Cluj-Napoca, Romania; ionut.anghel@cs.utcluj.ro

**Keywords:** whale optimization algorithm, cloud–edge offloading, smart grid, energy efficiency, directed acyclic graph

## Abstract

As IoT metering devices become increasingly prevalent, the smart energy grid encounters challenges associated with the transmission of large volumes of data affecting the latency of control services and the secure delivery of energy. Offloading computational work towards the edge is a viable option; however, effectively coordinating service execution on edge nodes presents significant challenges due to the vast search space making it difficult to identify optimal decisions within a limited timeframe. In this research paper, we utilize the whale optimization algorithm to decide and select the optimal edge nodes for executing services’ computational tasks. We employ a directed acyclic graph to model dependencies among computational nodes, data network links, smart grid energy assets, and energy network organization, thereby facilitating more efficient navigation within the decision space to identify the optimal solution. The offloading decision variables are represented as a binary vector, which is evaluated using a fitness function considering round-trip time and the correlation between edge-task computational resources. To effectively explore offloading strategies and prevent convergence to suboptimal solutions, we adapt the feedback mechanisms, an inertia weight coefficient, and a nonlinear convergence factor. The evaluation results are promising, demonstrating that the proposed solution can effectively consider both energy and data network constraints while enduring faster decision-making for optimization, with notable improvements in response time and a low average execution time of approximately 0.03 s per iteration. Additionally, on complex computational infrastructures modeled, our solution shows strong features in terms of diversity, fitness evolution, and execution time.

## 1. Introduction

The adoption and incorporation of Internet-of-Things (IoT) technologies in different economic sectors has led to the production of significant volumes of data, with high velocity and heterogeneity challenging the management processes requiring near real-time monitoring and control [1]. In the smart energy grid, smart metering devices and the prospect of small-scale renewable integration generate data processing and decision-making challenges for ensuring security in energy delivery and balancing the demand with the supply [2]. By connecting many IoT devices, a large quantity of data are fed and need to be considered in energy management processes that may be controlled and coordinated from a central location in the cloud [3].

However, in such scenarios, the entire data flow transfer to the cloud proves inefficient in terms of response time and bandwidth, a viable option being the offloading of computational tasks towards the edge [4]. It is facilitated by recent advances in edge computing and IoT technologies that have further expanded and decentralized the cloud paradigm with edge and fog resources to ensure the integration of computational capabilities closer to data sources [5]. Edge nodes with enough processing capacity are deployed to analyze energy IoT data and enable quicker decision-making processes to enhance the optimization of various collaborative and decentralized subsystems, allowing for efficient local energy management that contributes to the central grid objectives [6].

The edge computing paradigm enables offloading computational tasks from the cloud context closer to edge devices rather than only relying on the cloud for data processing. Such strategies can improve the smart grid operation, facilitating near real-time decision-making as it reduces the latency and usage of data network resources while addressing pressing issues related to the wide-area network capacity in transferring large quantities of data towards the cloud [7]. In addition, offloading computation reduces risks associated with data privacy and security breaches during data transit to the cloud. Processing sensitive information locally at the edge enhances data security and regulatory compliance, a vital consideration in smart grid systems where privacy and security are required [8]. Moreover, it enhances scalability, enabling distributed computing across interconnected devices and fostering dynamic and responsive systems, a key requirement for accommodating the evolving needs of smart grid operations. Thus, edge computing can play a significant role by reducing operational costs associated with cloud usage and optimizing resource allocation while minimizing data transfer overheads, which can make the smart grid decentralization and energy transition more cost-efficient and sustainable in the long run [6,9].

Even though the computational task offloading towards the edge brings significant benefits within smart grid contexts, from a decision-making perspective, determining where it is optimal to offload a task from the cloud node to the fog or edge nodes presents a significant challenge. The vast search space involved in evaluating various factors such as data volume, computational requirements, network conditions, and resource availability makes it difficult to identify efficient decisions within a limited timeframe. Heuristic methods can be employed to effectively model the optimization problem, leveraging on balancing the search space exploration and exploitation tailored to the unique requirements of smart grid operations [10,11]. Swarm-based algorithms are effective in addressing large and complex search spaces with multiple local optima. This is especially relevant in edge offloading, where there are various criteria to consider, such as resource availability, latency, and response time [12]. They are also effective for optimization problems in smart grid decentralization, such as computation resources allocation and scheduling, where traditional methods may struggle to find optimal solutions [13]. Incorporating heuristic algorithms into the decision-making process for edge offloading can boost the performance and efficiency of edge–cloud systems, characterized by rapid decision-making and a minimal use of computational resources [14].

In this paper, we address the above-presented challenges related to offloading decision-making by leveraging whale optimization to facilitate the optimal selection of edge nodes for the execution of virtualized tasks. We use directed acyclic graphs (DAGs) to model the spatial and logical dependencies of the computational nodes and the data network links, as well as the smart grid energy assets and energy network organization. The model foresees four different layers built one on top of the other: the physical smart grid infrastructure layer featuring the assets that are consuming or producing electricity, the edge node layer comprising computational devices closer to the energy assets, the fog device layer featuring intermediate nodes positioned between edge devices and centralized cloud servers in a network infrastructure, and the cloud layer. The constructed model facilitates more efficient navigation in the decision space by considering the inherent structure, nodes’ physical capabilities, and network dependencies within the computational continuum. On top of the graph model, we map and adapt the whale optimization algorithm (WOA) [15] to explore solution spaces and accelerate convergence toward optimal solutions. The offloading decision variables are represented as a binary vector, where each index corresponds to a specific computational node, and the total number of nodes determines the vector’s dimension. A fitness function is then defined to assess the quality of candidate solutions (whales) within the tasks’ offloading search space. This function is constructed based on components derived from the DAG, such as the round-trip time (RTT) between cloud and edge nodes, as well as the Euclidean distance between the requested task and the available computation resources of each node. The three primary phases—encircling, exploitation, and exploration—are tailored to address the task offloading problem. We integrate the enhancements proposed in [3], including the feedback mechanism, the nonlinear convergence factor, and the inertia weight coefficient. These adjustments facilitate the adaptive exploration of offloading strategies, ensuring effective resource utilization across the computational continuum while mitigating premature convergence to suboptimal solutions. As a result, the WOA coupled with the graph model enables informed and optimal edge-offloading decision-making, thereby enhancing system performance and efficiency.

The novel contributions of this paper are:

- A DAG model for spatial and logical dependencies of computational nodes, data network links, smart grid energy assets, and an energy network organized in four layers to enable efficient edge-offloading decision-making in a smart grid.
- A whale optimization algorithm adaptation for edge offloading, using binary decision variables for mapping workload to computational resources and a fitness function based on RTT and distance between tasks and available resources.
- Enhancements, including a feedback mechanism, nonlinear convergence factor, and an inertia weight coefficient, to efficiently explore offloading strategies in the solution space and avoid premature convergence.
- Evaluation of a smart grid scenario considering the offloading of energy balancing service with energy and data network constraints, necessitating fast decision-making for optimization.

The rest of the paper is organized as follows. Section 2 presents the related work on swarm-based heuristics applications for edge-offloading and orchestration decision-making. Section 3 provides an overview of the proposed solution focusing on computational continuum resource modeling and whale optimization. Section 4 presents the obtained results considering the smart grid-driven scenario and service, Section 5 discusses the results, and Section 6 concludes the paper and presents future work.

## 2. Related Work

Metaheuristic optimization algorithms play a pivotal role in making edge–fog–cloud offloading decisions due to their ability to explore complex solution spaces, adapt to changing conditions, and optimize resource allocation [16]. These algorithms offer robust solutions against the local optimum, ensuring that the global optimal solution is achieved. Their parallel and distributed nature enables scalable optimization, which can offer promising solutions for near-to-real-time decision-making in smart grids [13]. Due to their properties, they can be utilized to effectively allocate and oversee computational tasks across edge devices, considering factors like resource availability, latency, and response time for building edge-offloading decision-making processes with improved performance and efficiency. They can be used to find a close-to-optimal solution in complex solution spaces where traditional methods can hardly be applied.

The whale optimization algorithm (WOA) is a technique that emulates the communal hunting patterns of humpback whales, specifically drawing inspiration from their bubble-net feeding method [17]. It gained notoriety for different research approaches to solving optimization problems [18] leading to specific variations, improvements, and hybridization solutions tackling its weaknesses such as local optima trapping, exploration and exploitation imbalance, and low population diversity [19]. The metaheuristic algorithm is adaptable and is suitable for diverse smart grid scenarios and applications, which often vary greatly and demand flexibility and the ability to accommodate changing resource availability.

In the edge–fog–cloud domain, several approaches have tried to use or improve the standard WOA for resource allocation and job scheduling. Sing et al. [20] apply the WOA to develop a fog computing resource allocation algorithm for IoT applications. The approach initially uses a task classification and buffering technique based on dynamic fuzzy c-means clustering to classify tasks and employ the least slack time scheduling for parallel virtual queues. Then, task offloading and optimal resource allocation are performed, to decide task-offloading strategies and optimize resource allocation among fog nodes using the WOA to ensure enhanced throughput. The authors report a decrease in energy consumption and an increase in performance compared to a multi-objective monotonically increasing sorting-based algorithm. Mangalampalli et al. [15] elaborate on the task scheduling problem in cloud computing, emphasizing energy consumption and power cost reduction in data centers. Their approach utilizes the WOA to schedule tasks, mapping them to suitable virtual machines (VMs) considering task computations and VM priorities. By integrating the WOA, the proposed approach focuses on boosting energy efficiency and cutting down power expenses within cloud computing settings. Goyal et al. [21] present a method for optimizing resource allocations, reducing energy consumption in cloud infrastructure. Their approach employs a population of search agents that undergo fitness evaluation and iterative refinement to converge toward optimal solutions. Using a randomized search strategy, the algorithm dynamically adjusts agent positions based on fitness evaluations. The WOA plays a crucial role in achieving efficiency in cloud environments by balancing load, energy efficiency, and resource scheduling optimization and features better results when compared with particle swarm optimization (PSO), cat swarm optimization (CSO), BAT, and the cuckoo search algorithm (CSA). Abdel-Basset et al. [22] describe an enhanced version of the WOA for allocating dependent tasks in multi-processing systems, aiming to minimize energy consumption and makespan. It employs dynamic voltage and frequency scaling to reduce energy consumption in processing cores. Using specialized discretization methods and crossover operations, the technique generates high-quality candidate schedules while considering task dependencies. Its load-balancing strategy effectively reduces the load on heavy cores, leading to decreased static energy consumption. Yang et al. [23] designed an algorithm utilizing the WOA to tackle task allocation in IoT networks. By mimicking whale behaviors, it identified optimal positions for task allocation, promising energy efficiency and low-latency communication. The algorithm efficiently allocated tasks by evaluating optimal path nodes, assigning allocation abilities, and ensuring effective execution, encompassing both local and global processes for IoT optimization. Yaser et al. [24] introduce an algorithm designed to deploy services in fog environments in a way that emphasizes both reliability and energy efficiency, utilizing the WOA. Its objective is to minimize total power consumption while considering resource usage and traffic transmission among different services located on fog nodes. The algorithm includes reliability limitations as constraints in the optimization model. It executes update procedures for both exploration and exploitation, integrating additional unbiased updates by leveraging random whale positions for exploration and employing specialized updates for exploitation. Samoilenko et al. present the whale optimization method as a solution for task-offloading issues in cloud–fog environments [25]. This method utilizes the WOA for real-time, dynamic decision-making to improve quality-of-service aspects like execution delay and energy usage. It uses a group of potential solutions, depicted as whales, to optimally resolve the complex task-offloading challenge. In [26], an IoT service deployment solution in a fog infrastructure is proposed that uses the WOA to determine an efficient service placement plan. QoS requirements of IoT services and fog node capabilities are used as inputs, while throughput and energy consumption are considered in the objective functions. The evaluation shows that better resource usage, reduced service delay, and energy consumption can be obtained compared with other metaheuristics.

To overcome some of the WOA issues, researchers have tried to hybridize it with other algorithms while targeting edge–fog offloading in smart grid problems. Saoud et al. [27] propose a hybrid algorithm combining WOA and BAT methods, integrating BAT within the WOA’s search space and implementing a condition-based location update for each search agent. This approach aims to enhance the WOA’s exploration capabilities, mitigating the risk of local optima and addressing convergence challenges. Their combined strengths contribute to effective load balancing for scheduled resources in smart grids. The WOA-BAT hybrid algorithm emerges as a robust solution for optimizing smart grid operations, leveraging the complementary features of both the WOA and BAT. Amit et al. [28] focus on solving task scheduling problems in cloud environments through a hybrid metaheuristic approach. The authors highlight the limitations in the standard WOA related to solution diversity, convergence speed, and exploration–exploitation trade-off and propose h-DEWOA, integrating enhancements like chaotic maps for improved exploration, opposition-based learning for solution diversity, and differential evolution for enhanced exploration. A dedicated mechanism balances the fitness function to ensure a trade-off between exploration and exploitation. Kang et al. [29] present an intelligent hybrid whale optimization algorithm for multi-objective task selection in edge computing. It optimizes task selection by considering execution time and economic profits, addressing uncertainty with a fuzzy function. The algorithm enhances task selection by handling five interactive constraints and integrating strategies from the WOA to overcome local optima. Evaluation using synthetic datasets shows superior performance compared to the traditional WOA, as demonstrated by diversity metrics and hypervolume assessments. Huang et al. [30] enhance the WOA-based multi-objective algorithm for mobile edge computing offloading utilizing the gravity reference point method. The proposed technique enhances solution diversity, enabling trade-offs between time and energy consumption. Its improved diversity leads to enhanced convergence and optimal solutions outperforming traditional approaches. Feng et al. [31] hybridize grey wolf optimization (GWO) and the WOA to tackle computation offloading optimization in IoT and mobile edge computing systems. Their model incorporates three normalized targets for detailed offloading strategy evaluation, ensuring equal importance across factors. By considering suboptimal solutions, the algorithm aims for enhanced performance, seeking the most optimized value for the function. Anoop et al. [32] merge the differential evaluation with the WOA to optimize edge-offloading tasks. This hybrid approach addresses the shortcomings of traditional heuristic algorithms by leveraging the exploratory strength of the WOA and the detailed search efficiency of a differential evaluation. Inspired by the humpback whales’ spiral hunting technique, this algorithm enhances offloading strategies, leading to reduced energy use and quicker response times. In [33], the authors address the task scheduling problem in fog environments formulated as an integer linear programming optimization model that focuses on time and energy consumption efficiency. They propose a combination of the WOA, opposition-based learning, and chaos theory for solving the optimization problem. In the initialization phase of the WOA, opposition-based learning is applied to enhance diversity. Additionally, chaos theory is incorporated into the WOA to minimize the effects of random movements and steer more accurately toward the optimal solution. Lin et al. [34] approach smart grids’ optimal energy management problem by proposing a DAG-based framework to secure the data transactions above cloud–fog infrastructures and an agent-based approach to allow nodes to make a consensus. The WOA is used to solve the optimization problem for minimizing the grid network power loss of neighboring agents.

As analyzed above, the decision-making for computational tasks’ edge offloading in smart grids involves balancing factors like latency, energy efficiency, resource optimization to ensure optimal performance and reliability of the grid. Energy services’ tasks that require low latency and deal with large data volumes should be offloaded to edge devices. Whale optimization supports dynamic decision-making to adapt to changing conditions in the grid, such as fluctuations in energy demand or the availability of renewable energy sources. Additionally, the WOA is a good choice as it can be adapted to balance exploration and exploitation of the search space, while aiming for optimization considering both computational and data network constraints as well as the energy network and smart grid services constraints.

Furthermore, we map and adapt the original WOA algorithm to the specific problem of computational tasks’ offloading towards the edge by modeling the solutions as binary decision variables storing the workload distribution and defining a fitness function based on the RTT and distance between tasks and available resources. As the energy grid and computational continuum infrastructures can be complex and difficult to consider in decision-making, we consider a DAG model to represent the decision variables and provide a comprehensive view of the dynamics. Our work considers several enhancements to the whale optimization algorithm, including a feedback mechanism, nonlinear convergence factor, and inertia weight coefficient [21,27]. These enhancements facilitate a more efficient exploration of offloading strategies within the solution space, thereby avoiding premature convergence and improving overall performance. Finally, we evaluate our approach in the context of a smart grid scenario, focusing on the offloading of energy balancing services while considering energy and data network constraints. We highlight the need for fast decision-making to optimize energy utilization and ensure grid stability, demonstrating the practical relevance and effectiveness of our proposed techniques.

## 3. Materials and Methods

In this section, we present the edge-offloading decision-making process focusing on energy and computational resource modeling and the WOA adaptation and implementation for this specific case.

### 3.1. Energy and Computational Networks Resources

We model the computing continuum as a connected computationally directed acyclic graph:

(1) G=(V,E)

where V represents a finite set of vertices representing computational nodes available in edges, the fog, or the cloud, and E is a set of ordered pairs of vertices, representing directed edges that model the data network links between the computational nodes. For each type of node and edge, we define the relevant properties as graph annotations to enable the whale optimization algorithm to reason on top and to take optimal task-offloading decisions.

In the graph, we define layers considering the overlaps and interactions among the data network and computational resources and the smart grid energy network: the physical energy asset layer, edge node layer, fog node layer, and cloud node layer. Each layer fulfills distinct tasks crucial to the overall computational ecosystem of the smart grid. The DAG provides a formal representation to express the interactions and dependencies among these layers, defining the roles and contributions for each layer.

The *energy asset layer* models the devices and resources of the smart grid that generate, store, or consume electrical energy. It consists of renewable-energy generators (e.g., solar panels, wind turbines), conventional power plants, energy storage systems, electric vehicle power stations, and residential prosumers. The energy assets play a fundamental role in the smart grid management, making the integration of renewable energy sources possible, participating in the demand response programs, and providing energy services to enhance resilience and reliability for the overall smart grid. To effectively contribute to closer-to-real-time management and control, the management tasks should be offloaded to nodes closer to their physical or logical locations.

**Figure 1:**
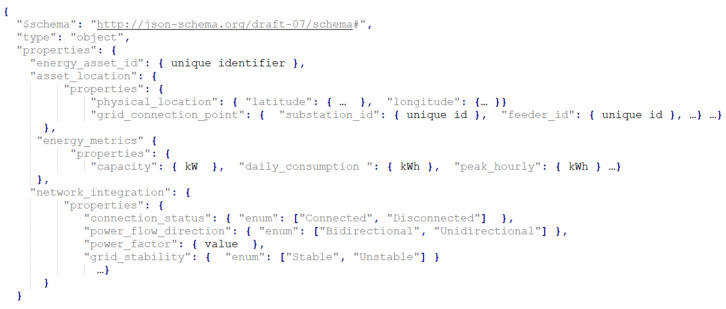
Energy assets’ node annotation.

The annotation model for this type of node is presented in Figure 1. It is organized into three main sections. The first section contains general information about the assets, such as the unique identifier and the physical position of the asset using longitude and latitude coordinates. The second section describes energy metrics for the assets, detailing their energy-related behavior including the maximum capacity of the asset, average daily energy consumption, and peak hourly energy consumption.

The third section provides details about the connection point to the electrical grid, including the identifiers of the substation and feeder the asset is connected to, and integration details of the asset with the electrical grid, such as the connection status, direction of power flow, power factor, etc.

The *edge node layer* models the edge nodes as computational units situated at the edge of the data network, typically near energy assets within the smart grid infrastructure. They are critical points for aggregating, analyzing, and transmitting data generated by energy assets, and executing tasks such as closer-to-real-time control and the optimization of grid operations. The edge nodes play a role in improving the responsiveness and reliability of the energy network and efficiency of the data and computational network as they enable the local energy data processing, reducing the latency associated. In the DAG representation, edge nodes are represented as vertices V, while the edges E in our case represent the connection with one or more energy assets of the smart grid.

**Figure 2:**
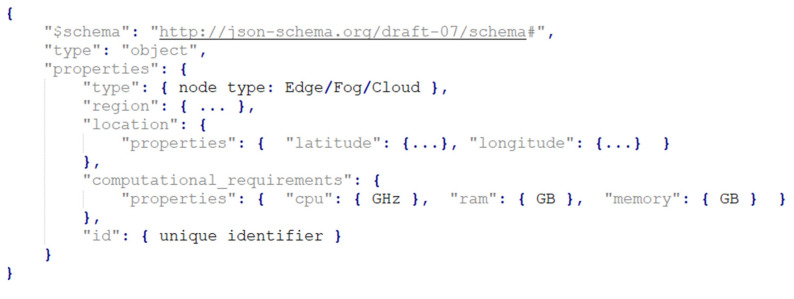
Edge layer node annotation.

The annotation model for the edge nodes is presented in Figure 2. The model contains two main sections to ensure clarity and interoperability within the network environment, both being valuable information for making proper offloading decisions. The first section represents the type of node, indicating its role within the network, the geographical region where the node is located, aiming for effective resource management, the precise latitude and longitude coordinates, facilitating analysis and positioning within the smart grid infrastructure, and the unique identifier. The second section contains the computational resources required by the node, including CPU speed, RAM size, and storage capacity.

**Figure 3:**
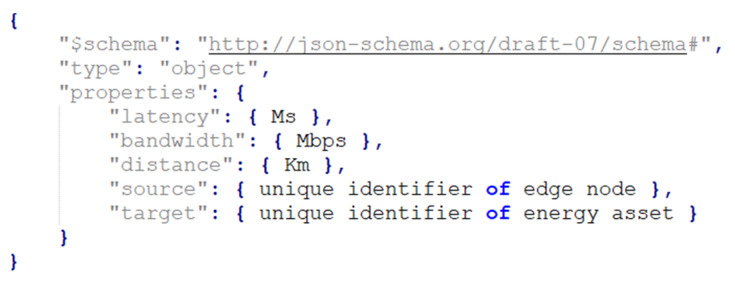
Links’ annotation model.

The annotation model for data links between the edge nodes and energy assets is illustrated in Figure 3. The model represents the network connectivity parameters between an edge node and an energy asset. It includes properties such as latency, bandwidth, and distance, to assess the performance and positioning within the network connection. Latency measures the time delay in data transmission, bandwidth specifies the maximum data transfer rate, and distance indicates the physical separation between the entities. Additionally, unique identifiers for both the edge node and the energy assets are included to express the communication within the network infrastructure.

*The fog layer models nodes* that are strategically positioned between edge and cloud layers to facilitate edge node orchestration for data processing and offloading tasks from the cloud. Their proximity to both edge nodes and energy assets enables them to host critical services such as AI/ML tasks for energy management which may enhance grid efficiency and reliability. Represented as vertices in the DAG model, fog nodes maintain a vital role in balancing local and cloud-based processing on one side and ensuring fast decision-making for smart grid optimal operation on the other side.

The annotation model for fog nodes follows the same structure as for edge nodes, albeit with significantly higher values for properties like CPU speed, RAM size, and storage capacity. These improved capacities underscore the enhanced computational power of fog nodes and their essential role in supporting diverse tasks within the smart grid. Additionally, the annotation model for data links between fog and edge nodes follows a similar structure to the edge node-to-energy asset connection model. In this context, data links between fog and edge nodes show substantially higher values for properties like bandwidth and lower latency, highlighting the superior data processing capabilities and faster communication speeds inherent to fog nodes. These distinctions are important in optimizing network performance, enhancing data transmission efficiency, and ultimately facilitating the smooth operation of the smart grid infrastructure.

*The cloud layer* models nodes that represent a centralized computing environment situated remotely from the grid’s edge, offering extensive computational resources for tasks like data analytics, machine learning algorithms, and long-term planning. Unlike edge and fog nodes, cloud nodes provide robust computing capabilities, symbolized as vertices V in the DAG model, with edges E connecting them to fog nodes. The annotation model for cloud nodes maintains a structure like that of edge Nodes, featuring properties like CPU speed, RAM size, and storage capacity, which typically have significantly higher values than edge and fog nodes. Distinct differences emerge in values for properties like bandwidth, latency, and distance in the annotation model for data links between cloud nodes and fog nodes. These differences underscore the remote nature of cloud nodes and their reliance on high-speed, long-distance communication links to interact with fog nodes at the network edge. These distinctions are crucial for optimizing network performance and facilitating efficient data transmission within the smart grid infrastructure.

### 3.2. WOA-Based Offloading Technique

**Figure 4:**
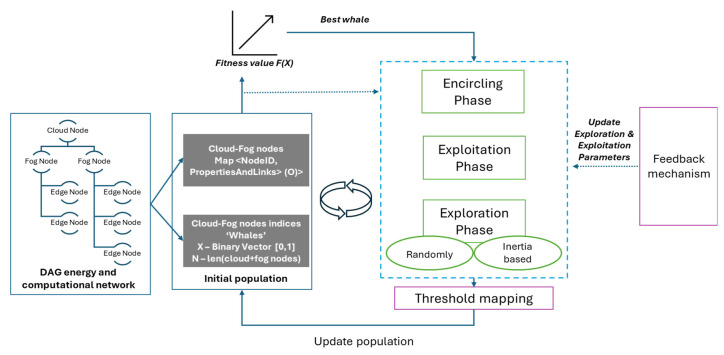
Data flow for the offloading decision.

Figure 4 illustrates the data flow of the offloading decision process. In our approach, each whale represents the nodes within the computational continuum resources deployed on top of the smart grid being modeled using the DAG nodes’ annotation model (denoted as M). This model encapsulates crucial computational characteristics necessary for making informed offloading decisions, including geolocation and communication links, as specified by the DAG links’ annotation model (denoted as N). The cloud fog nodes’ mapping (denoted by O) stores the nodes’ unique identifiers and their properties and links and is essential for the result of the offloading decision.

In the offloading process, cloud nodes should identify potential fog/edge nodes based on factors such as round-trip time (RTT) and computational compatibility with the task requirements. This entails evaluating the distance, both in terms of network latency and computational capability, between the tasks and the available nodes.

The offloading decisions’ variable employs a whale representation as a binary vector:

(2) $$X=\left[x_{1},x_{2},\ldots x_{nodes}\right],\;x_{i}=\{0,1\}$$

where the nodes dimension represents the total number of computational nodes and each index in the vector identifies a specific node. The indexes marked with 0 represent the nodes with no computational tasks offloaded or assigned, while the nodes marked with 1 represent the nodes selected for tasks offloading based on the considered criteria. The decision-making and optimal selection of nodes are performed by using an adapted version of the hybrid modified whale optimization algorithm [21].

A fitness function is defined to evaluate the quality of candidate solutions (whales) in the search space of tasks’ offloading. We incorporated into the fitness function constraints associated with RTT between cloud and edge nodes and with the Euclidean distance D between the task requested and node available computation resources:

(3) $$F\left(X\right)=\underset{\mathrm{RTT},D}{\min}\left(X\right)$$

In this way, the function penalizes solutions that violate these types of constraints, thereby directing the search towards feasible and optimal solutions.

Minimizing the *RTT* is crucial for near real-time and time-sensitive smart grid management applications in computational continuum offloading scenarios. The *RTT* represents the duration required for data to traverse from an edge device to a cloud one and subsequently return:

(4) $$RTT=\left(P_{d}+T_{d}+\frac{l}{1000}\right)*1000$$

where $P_{d}$ is the propagation delay representing the duration for a signal to traverse the distance between the sender and receiver, computed as the ratio of distance and the speed of light, $T_{d}$ is the transmission delay representing the time required to transmit a packet from the host to the transmission medium and l is the transmission delay or latency.

The parameters derived from the DAG annotation model constructed for the edge–fog–cloud network of resources are converted to their respective units (i.e., bandwidth in bits per second, distance in meters). The propagation and transmission delays are computed and combined with the latency, all expressed in seconds. Finally, the cumulative delay is converted back to milliseconds before being returned as the result.

Computational resources such as CPU, storage, and RAM are integral in facilitating the offloading of specific tasks from the cloud and fog to the edge. The availability of these computational resources plays a crucial role in determining the feasibility of offloading. We used the Euclidean distance to determine the matching between the workload tasks’ computational resources’ requirements and the node available resources considering *CPU*, storage (*HDD*), and *RAM* as its dimensions.

(5) $$D=\surd ({task_{CPU}-Node_{CPU})}^{2}+({task_{RAM}-Node_{RAM})}^{2}+({task_{HDD}-Node_{HDD})}^{2}$$

This indicates the dissimilarity between the computational requirements of the tasks and edge nodes and the computational capacity of the edge node, guiding the decision-making process for offloading.

In the whale optimization algorithm (WOA), as introduced in [15,20], three main phases are defined: the encircling phase, the exploitation phase, and the exploration phase.

During the encircling phase, the whales coordinate towards their prey, which represents the optimal edge-offloading solution. Thus, the offloading agents X update their positions using the following relations [20]:

(6) $$D^{\to }=|C^{\to }\;*\;X^{\to \prime }(t)-X^{\to }(t)|$$

(7) $$X^{\to }(t+1)=X^{\to \prime }(t)-A^{\to }\;*\;D$$

where t represents the current iteration of the algorithm, $X^{\to \prime }(t)$ is the position in the search space of a potential edge-offloading solution determined by virtually placing the task on an available node. $A^{\to }$ and $C^{\to }$ are coefficients of the vectors which are randomly generated with values in the range [0, 1]. Those are used to control the exploration of potential offloading decisions across the available nodes.

The exploitation phase represents the bubble-net method for attacking the prey and is based on two steps: the shrinking encircling and the update of the spiral position. The shrinking encircling involves randomly generated values from the $A^{\to }$ vector in the range [−a, a], where a decreases linearly. This reduces the range of exploration, focusing the search on potentially optimal offloading decisions.

In the spiral updating position step, the algorithm computes the distance between the current offloading decision $X^{\to \prime }\;(t)$ and potential target location $X^{\to }\;(t)$ representing the prey [20]:

(8) $$D^{\to \prime \prime }=X^{\to \prime }\;(t)-X^{\to }\;(t)$$

Then, it determines the updated position $X^{\to }\;(t+1)$, considering factors such as the distance of the target and the shape of the spiral path:

(9) $$X^{\to }\;(t+1)=D^{\to \prime \prime }\;*\;bl\;*\;cos\;(2l)+X^{\to \prime }\;$$

where the shape of the spiral path is defined by constants b and l, randomly generated in the range [−1, 1].

In our offloading decision process, the shrinking encircling and spiral updating position steps are linked to the adjustment of offloading decisions based on minimizing factors such as the RTT (relation (4)) and computational distance between tasks and nodes (relation (5)).

In the exploration phase, the process is analogous to the behavior of whales searching for prey. The solution agents $X^{\to }$ explore potential new solutions $X^{\to }\;(t+1)$ randomly based on their current positions [20]:

(10) $$X^{\to }\;(t+1)=X^{\to }\;rand-A^{\to }\;*\;D\;$$

where $X^{\to }$ rand represents a randomly selected position vector from the population in the current iteration, $A^{\to }$ is the coefficient vector that controls the step size for updating the offloading decision, and $D^{\to }$ is the difference vector between the current position and the best solution found so far.

The WOA takes as inputs the population size, maximum number of iterations, and initial values for the parameters a, A, and C. Initially, the offloading agents, representing the search agents, are randomly generated, and their fitness values are computed. The best search agent is identified based on its fitness value.

We incorporated the enhancements introduced in [21], specifically the feedback mechanism, the nonlinear convergence factor, and the inertia weight coefficient to facilitate the adaptive exploration of offloading strategies, ensuring an effective utilization of resources across the computational continuum while avoiding premature convergence to suboptimal solutions.

The feedback mechanism ensures that if the optimal offloading decisions remain unchanged for a certain number of iterations, the algorithm introduces randomness to prevent stagnation and escape local optima in the offloading decisions. This improves the overall convergence accuracy of the algorithm for making proper edge-offloading decisions.

The convergence factor $A^{\to }$, defined in relation (7), is based on the current iteration t and the maximum number of iterations $t_{max}$. Unlike the traditional linear decrease of $A^{\to }$, the nonlinear decline introduced by [27] aims to determine the number a in the interval [−a, a] in which $A^{\to }$ takes values as:

(11) $$a=2+2\;*\;cos\;(2\;x\;(1+t_{max}))$$

This enhances convergence speed while ensuring a balance between exploration and exploitation. As a result, the algorithm can efficiently explore the search space of potential offloading solutions across edge and fog nodes.

The updated position of a solution $X^{\to }\;(t+1)$ is determined by considering an additional inertia weight coefficient [21] w:

(12) $$X^{\to }\left(t+1\right)=w*X^{\to \prime }\left(t\right)-A^{\to }\;*\;D$$

The weight coefficient is determined like in particle swarm optimization based on the current iteration t and the maximum number of iterations $t_{max}$:

(13) $$w=0.5+0.5*({\frac{t}{t_{max}})}^{2}$$

In the offloading decision, it adjusts the impact of historical offloading decisions on the current search direction, enhancing the accuracy and speed of the search process. It gradually increases from 0.5 to 1 over iterations, enhancing accuracy and speeding up convergence. This adaptation aids in fine-tuning the exploration and exploitation trade-off during the optimization process.

On top of the X vector result, our approach identifies the most suitable node for making the offloading decision thus tasks associated with the candidate node for offloading are shifted to the best target node. The result includes information such as the total number of nodes, and the number of nodes selected for offloading. The vector of nodes to offload contains specific details for each node, such as the best offloading target and relevant distance metrics.

Algorithm 1 shows the WOA algorithm applied to the edge offloading in a smart grid. It takes as input the DAG model describing the energy and computation infrastructure, the tasks to be relocated with their requirements, and WOA parameters such as the number of iterations, size of the population, etc. First, the initial population is generated by considering the DAG model and tasks to be relocated and the fitness function is evaluated on each member to determine the best one using RTT and computational requirements distance as criteria (see lines 1–3). The best member of the population constitutes the prey in the following whale hunting steps. At each iteration, whales explore the search space and update their positions based on the exploration and exploitation equations (lines 6–14). Similarly to [21], we integrated a feedback mechanism to prevent stagnation and escape local optima during optimization (lines 19–20). It introduces randomness to the search process if optimal offloading decisions remain unchanged. This ensures a continued exploration of the solution space for node selection in offloading decisions, where multiple local optima may exist. The adaptive feedback mechanism considers the RTT and computational distance among tasks and node resources. The parameters are dynamically adjusted based on these factors to prioritize solutions, minimizing both resource usage and communication latency by balancing exploration and exploitation through the adaptation process (lines 10–15). To adapt the WOA to the binary problem of edge offloading in a smart grid, we used a threshold-based mapping technique. After each algorithm iteration of the algorithm, the continuous solutions are converted into binary solutions using thresholds (see line 23).
**Algorithm 1:** WOA for computational offloading decision-making in a smart gridInputs: $DAG_{model}$—energy and computational nodes and links, population size $P_{size}$, maximum number of iterations $t_{max}$, WOA parameters (a, A, C), tasks—workload to be relocated Outputs:
$X_{sol}$—Best offloading decision solution Begin 1. $P=\left\{X_{i}\right\}=genInitialPopulation(DAG_{model},\;tasks$) 2. Foreach $X_{i}$ determine the fitness value $F\left(X_{i}\right)$ 3. Select $X_{best}=min\;F\left(X_{i}\right)$ 4. Set initial values for $c,\;A,\;D,\;t_{max},\;t=0$ 5. $while\left(t<t_{max}\right)\;do$ 6.   Foreach solution $X_{i}^{\prime }(t)$ in P, do 7.    Encircling phase: 8.      Calculate distance between $X_{i}^{\prime }\left(t\right)$ and $X_{best}\left(t\right)$ 9.      Determine position $X_{i}(t+1$) using $X_{i}^{\prime }\left(t\right)$ and $X_{best}\left(t\right)$ in relations (6) and (7) 10.     Exploitation phase: 11.        Determine position $X_{i}(t+1$) using $X_{i}^{\prime }\left(t\right)$ and $X_{best}\left(t\right)$ in relations (8) and (9) 12.     Random exploration phase: 13.        Explore new solutions $X_{i}(t+1$) using relation (10) 14.     Inertia-based exploration phase: 15.        Explore new solutions $X_{i}(t+1$) using inertia weight in relations (12) and (13) 16.   End Foreach 17.   Foreach $X_{i}\left(t+1\right)$, determine the fitness value $F\left(X_{i}\left(t+1\right)\right)$ 18.      Select $X_{best}$= $\min F\left(X_{i}\left(t+1\right)\right)$ 19.   If $X_{best}$ remains unchanged for several iterations do 20.      Update exploitation / exploration parameters 21.   *t* = *t* + 1 22. end while 23. Foreach $X_{i}(t+1)$, apply threshold mapping to convert continuous values to binary. 24. return $X_{sol}=X_{best}$ End

## 4. Results

**Figure 5:**
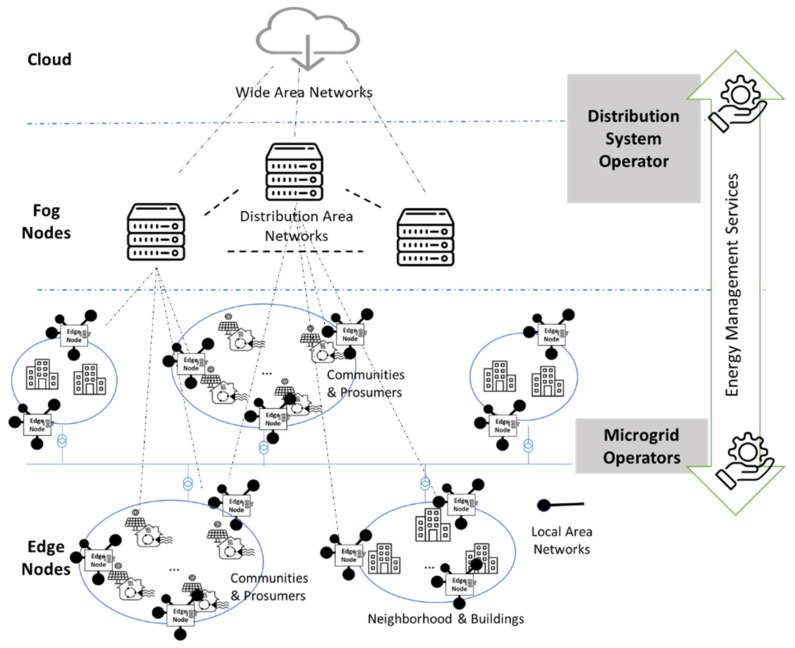
Edge–fog–cloud computational resources’ distribution for smart grid scenarios.

In this section, we evaluate the effectiveness of the proposed WOA technique in facilitating offloading within the computing continuum considering a smart grid scenario. Figure 5 presents an overview of the considered scenario for computational resources’ distribution over the smart grid for efficient delivery of energy services. The microgrid system operator (MSO), positioned closer to the edge nodes, oversees the distribution of electricity within a specific area or locality such as a neighborhood. It deals with the local coordination of energy assets part of the microgrid to ensure an efficient and reliable energy supply while optimizing performance and minimizing costs. It has a limited view of the assets and coordination problems outside the microgrid. The distribution system operator (DSO) oversees a larger geographical region, facilitating the flow of electricity among various microgrids. This includes tasks such as managing voltage levels, balancing loads, and ensuring the reliability and stability of the electrical grid within a city. To achieve this, the DSO utilizes a fog/cloud infrastructure to integrate monitored data from numerous energy assets and neighborhoods. This integrated information allows for optimal decision-making in response to supply fluctuations and grid conditions.

We considered an energy balancing service in a smart grid with both energy and data network constraints, necessitating fast decision-making for optimization. The energy balancing service monitors energy generation from prosumers, energy demand from consumers, and energy storage capacities. It should continuously adjust energy flow to ensure a balance between supply and demand in real time. Additionally, the data network has constraints such as bandwidth limitations for transmitting real-time energy data between sensors, meters, and control centers which generates latency in decision-making. Thus, the service response time is influenced by the time it takes to monitor the energy demand and supply ($T_{monitor}$) and transmit the data ($T_{transmision-data}$), decide $T_{decision}$ on power flow control actions, transmit the decision ($T_{transmision-decision}$), and enforce the action:

(14) $$T_{response}^{service}=T_{monitor}+T_{transmision-data}+T_{decision}+T_{transmision-decision}$$

In this case, edge offloading should enable faster data processing at the data network edge, allowing for a quicker analysis and decision-making as it decreases the latency and increases locality thus minimizing the data and decision transmission times. In the case of DSO, this means that the workload dealing with load balancing and grid stability could be offloaded onto edge nodes deployed at the microgrid level to act swiftly without relying solely on centralized fog/cloud servers. In this case, the aim is to reduce overall latency by processing data closer to the edge, thereby minimizing the round-trip time (RTT). By processing data locally at the edge, the need for transmitting data to remote fog and cloud servers is reduced, leading to shorter RTTs and thus faster service reaction time.

We started by modeling the energy and computational resources based on the defined scenario, using the DAG model described in Section 3.1. Subsequently, we utilized the WOA offloading technique presented in Section 3.2 to simulate and prompt offloading decisions aimed at minimizing service response times.

The *energy asset layer* comprised five communities of prosumers organized in microgrids with a total number of 125 prosumers and 23 consumers distributed per microgrid as described in Table 1. The prosumers may have photovoltaic (PV) solar panel systems for production and use their own consumption devices. Prosumers were classified into three categories based on their energy production capacity: low (up to 3 kW PV), medium (5 kW PV), and high (up to 7 kW PV). In the second, fourth, and fifth microgrids, some of the assets were only consumed without any local production on site. The surplus of energy could be stored in a centralized storage system at the level of each microgrid.

The *edge node layer* contained 20 edge nodes connecting to prosumers and microgrids defined in the energy asset layer. Their computational capacities’ range varied as presented in Table 2.

The *fog node layer* consisted of three microdata centers (DCs) with a total number of 12 fog nodes with links to the edge node layer. Micro DCs contain servers and virtual machines, which are close to the generation of data, in this case to the edge nodes layer. Within these micro DCs, we defined three capacity ranges to express the diverse computational capacities, as shown in Table 3.

The connections from the fog layer to the edge layer maintained a latency within the scope of 50 to 100 milliseconds, alongside a bandwidth varying from 10 to 500 megabits per second and distances ranging from 200 to 700 km.

The *cloud node layer* included three clouds from different regions, with a total number of 10 nodes with links to the fog nodes. These clouds could be in a faraway location from the micro DCs of fog nodes and devices of edge nodes. Table 4 shows the diverse computational capacities of the DC nodes. The connections from the cloud layer to the fog layer contained a latency within the scope of 50 to 110 milliseconds, alongside a bandwidth varying from 100 to 1000 megabits per second and distances ranging from 300 to 1000 km.

**Figure 6:**
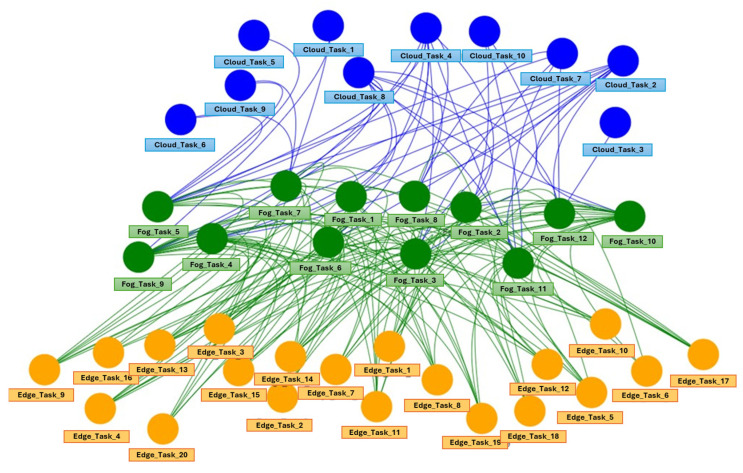
DAG for the described smart grid scenario.

The DAG model generated for the presented scenario is illustrated in Figure 6.

To make offloading decisions using the proposed WOA-based technique on top of the DAG, we set specific thresholds as shown in Table 5 to ensure that the balancing services’ response time request was met and minimized.

Table 6 presents the offloading computations results based on the WOA from cloud to fog nodes, and subsequently from fog to edge nodes. A noticeable improvement in response time was observed when comparing the distances and RTT before and after offloading, indicating enhanced performance. For instance, tasks initially assigned to cloud node ID 2 were offloaded to fog node ID 5, resulting in a decrease in both computational distance (6.07) and round-trip time (89.71 ms), bringing them comfortably under the thresholds. Similar improvements were observed across other offloaded tasks, such as those initially assigned to the cloud node with IDs 8 and 10, now processed more efficiently at the fog node with IDs 10 and 9, respectively. Furthermore, the offloading decisions from fog to edge nodes also contributed to the enhanced response time. Tasks originally handled by fog node IDs 8, 9, and 10 were efficiently processed at the edge node with IDs 17, 9, and 2, respectively, leading to reduced computational distances and round-trip times.

**Figure 7:**
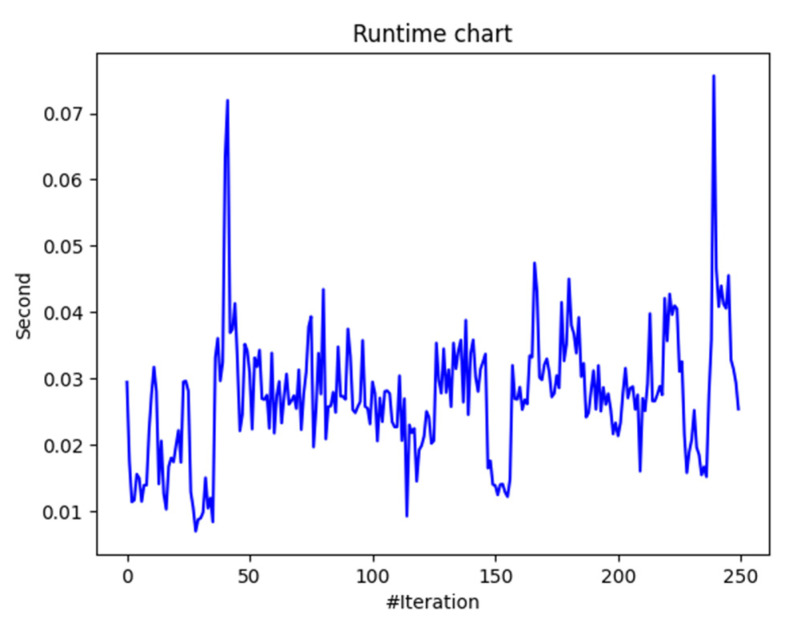
WOA offloading execution time.

In terms of the WOA for edge offloading, the convergence occurred after iteration 230, where the fitness value stabilized, suggesting the algorithm likely found near-optimal solutions for task offloading to the edge for the considered smart grid scenario. The average execution time remained at low values and was stable at 0.03 s per iteration for most of the WOA optimization process (see Figure 7). The spikes in the runtime chart at iterations 45 and 240, each taking 0.07 s, suggest the inference of other tasks executed in the background on the device where the offloading algorithm was run.

The time and space complexity of the developed edge-offloading algorithm is given by the number of iterations required by the WOA to converge to a near-optimal solution. It depends on the population size ($P_{size}$), search space dimensions, and convergence rate in the number of iterations ($t_{max}$). The search space dimension is determined by the number of computational nodes (|X|) available in the edge and fog layers and the number of tasks to be offloaded ($n_{tasks}$):

(15) $$O(P_{size}x|X|xn_{tasks}xt_{max})$$

Additionally, the space complexity of our implementation depends on the memory requirements for storing the vectors representing each element of the population (see relation (2)) and additional data structures used during the optimization process, which in our case were neglectable.

## 5. Discussion

To analyze the effectiveness of the proposed WOA for edge offloading, we varied the node distribution for the edge, fog, and cloud layers as shown in Table 7, and determined the impact on heuristic features such as diversity, fitness function evolution, exploitation vs. exploration, and execution time.

We used the diverse set of nodes and their respective computational capabilities to generate DAGs with different levels of complexity for two task-offloading scenarios. By strategically allocating tasks based on their computational requirements and data connection constraints, our solution sought to determine the optimal task-offloading allocation for specific scenario configurations.

The diversity feature measures the variety of the offloading solutions within the overall population of whales. The diversity is measured by calculating the Euclidean distance between each pair of solutions in the population to quantify the spread or distribution of edge-offloading solutions determined. A higher average value reflects a higher diversity and ensures that a wider range of potential solutions are generated, leading to a better exploration of the search space.

**Figure 8:**
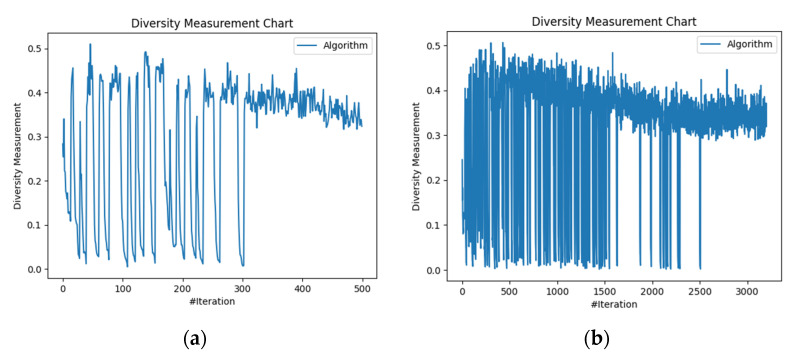
Diversity measurement variation with iterations and number of nodes: (**a**) scenario 1 and (**b**) scenario 2.

We analyzed diversity among solutions for the two generated scenarios aiming to determine the impact of increasing the number of nodes. In scenario 1, the diversity showed large fluctuations from 0.0 to 0.5, indicating a broad exploration of solutions (see Figure 8a). From the 300th iteration onwards, it stabilized between 0.3 and 0.4, suggesting convergence towards consistent solutions. By the 500th iteration, diversity settled at 0.35, indicating a moderate level of diversity among the solutions. In scenario 2, where we raised the number of edge and fog nodes as previously defined, only after the 2800th iteration did the diversity measurements stabilize, suggesting convergence towards consistent solutions (see Figure 8b). More computational nodes provide additional opportunities for solutions exploration and thus lead to a higher diversity initially. However, convergence towards consistent solutions takes longer (in terms of algorithm iterations) due to the increased complexity introduced by the additional edge/fog nodes.

**Figure 9:**
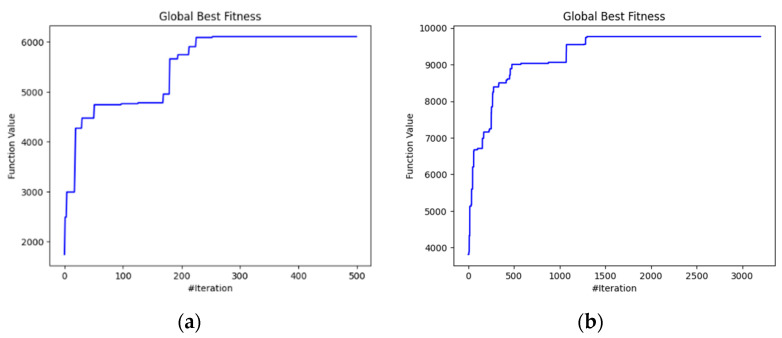
Global fitness evolution for different number of nodes: (**a**) scenario 1 and (**b**) scenario 2.

Next, we examined the global fitness evolution to analyze how effectively the WOA improved task-offloading solutions over time and how close it came to finding the optimal or near-optimal solution. In scenario 1, with a lower number of nodes (Figure 9a), convergence was reached after the 250th iteration without any spikes, indicating smooth improvement towards an optimal solution. In scenario 2 (Figure 9b), where more nodes were defined, convergence was achieved only after 1300 iterations, showing the nonlinear relationship between the number of nodes and convergence complexity. Even in this case the converge showed efficient improvement towards an optimal solution without encountering spikes.

**Figure 10:**
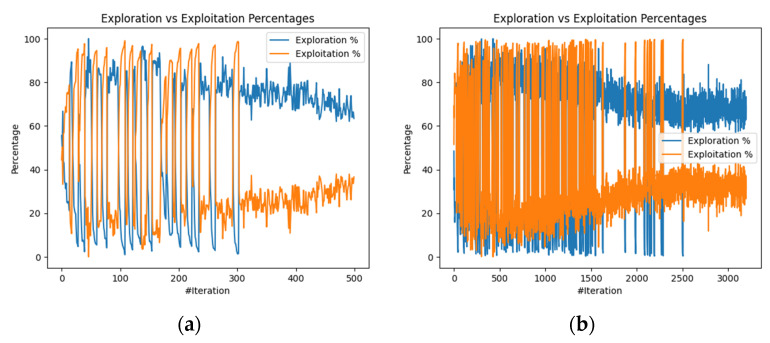
Balancing exploration and exploitation: (**a**) scenario 1 and (**b**) scenario 2.

The third aspect analyzed was how well the WOA balanced the exploration (diversification) and exploitation (intensification) of the search space during the edge-offloading decision-making process. When the exploration vs. exploitation percentages stabilize, it indicates that the algorithm has achieved a balance between exploring new solutions and exploiting known promising regions within the search space. This happened for the final iteration 500 for 50 edge nodes (Figure 10a) and from the 2700th iteration onwards for the 70-edge-node case (Figure 10b). The obtained balance results for the defined scenarios show that the proposed algorithm explored the search space sufficiently to avoid getting trapped in local optima while also exploiting promising edge-offloading solutions to converge towards the global optimum.

**Figure 11:**
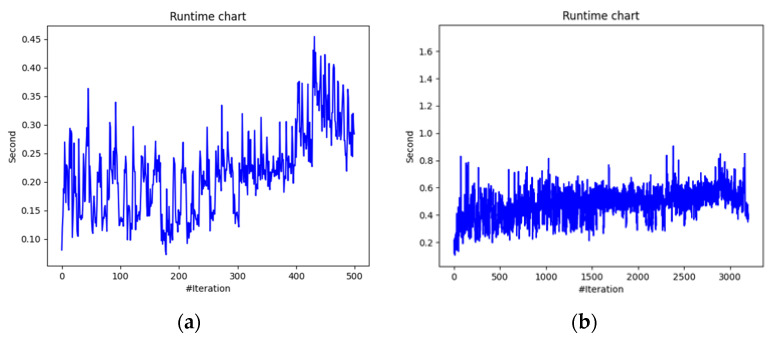
Tasks-offloading decision-making time: (**a**) scenario 1 and (**b**) scenario 2.

In terms of algorithm execution time, the chart in Figure 10 exhibits occasional spikes, suggesting concurrent processes were active. Despite these spikes, the average runtime remained consistent at 0.25 s per iteration for scenario 1 (Figure 11a). The maximum observed runtime occurred during the 400th iteration, reaching 0.45 s. For scenario 2 (Figure 11b), spikes persisted until the 2700th iteration, with an average runtime of 0.5 s.

## 6. Conclusions

In this paper, we adapted and applied the whale optimization algorithm for edge-offloading decision-making in a smart grid. We used a directed acyclic graph to model the dependencies of computational nodes, data network links, smart grid energy assets, and energy network organization using four layers: physical smart grid infrastructure, edge nodes, fog devices, and cloud. The whale optimization was implemented on top of the graph model to explore the solution space and efficiently converge toward optimal solutions. The offloading decision variables were represented as a binary vector, and a fitness function evaluated the quality of candidate solutions using components derived from the DAG, such as the round-trip time and the distance between the task requests and each node’s computation resources. We adapted the feedback mechanism, nonlinear convergence factor, and inertia weight coefficient to enable an adaptive exploration of offloading strategies, ensuring effective resource use and optimal edge-offloading decisions.

The evaluation results were promising, demonstrating that the proposed solution could effectively consider both energy and data network constraints for an energy-balancing service in the smart grid. The response time showed improvement when compared to distances and round-trip time (RTT) before and after offloading, indicating enhanced performance. The average execution time per iteration remained steady at approximately 0.03 s. Convergence was observed after iteration 230, as indicated by the stabilization of the fitness value.

Furthermore, when applied to complex computational infrastructures, our solution demonstrated robust characteristics such as diversity, fitness evolution, and efficient execution times. The increased number of computational nodes offered more opportunities for exploring solutions, resulting in higher diversity initially, but the convergence towards consistent solutions required more iterations. It effectively navigated the search space, avoiding local optima while capitalizing on promising edge-offloading solutions to converge towards the global optimum. Moreover, the execution time remained consistently low per iteration.

## Figures and Tables

**Table 1:** The prosumers and consumers on each microgrid for the energy asset layer.

Microgrid ID	No. Energy Assets	Production Capacity Prosumers			Consumers
		Low	Medium	High	
1	20	10	0	10	0
2	20	5	7	3	5
3	30	15	10	5	0
4	35	12	13	0	10
5	43	20	8	7	8

**Table 2:** Edge node layer’s distribution of devices.

# Connected Prosumers	# Edge Nodes	Low-Capacity Nodes (35 GHz CPU, 2 GB RAM, and 10 GB Storage)	Medium-Capacity Nodes (45 GHz CPU, 4 GB RAM, and 25 GB Storage)	High-Capacity Nodes (50 GHz CPU, 8 GB RAM, and 32 GB Storage)
30	5	2	3	0
45	4	2	1	1
25	3	3	0	0
25	8	5	2	1

**Table 3:** Fog node specification and edge layer connection.

# Connected Edge Nodes	# Fog Nodes	Low-Capacity Nodes (40 GHz CPU, 4 GB RAM, and 16 GB Storage)	Medium-Capacity Nodes (48 GHz CPU, 8 GB RAM, and 28 GB Storage)	High-Capacity Nodes (50 GHz CPU, 16 GB RAM, and 32 GB Storage)
7	4	2	2	0
8	5	2	2	1
5	3	1	2	0

**Table 4:** Distribution in terms of cloud nodes.

# Connected Fog Nodes	# Cloud Nodes	Low-Capacity Nodes (42 GHz CPU, 8 GB RAM, and 25 GB Storage)	Medium-Capacity Nodes (48 GHz CPU, 16 GB RAM, and 30 GB Storage)	High-Capacity Nodes (50 GHz CPU, 32 GB RAM, and 32 GB Storage)
2	2	2	0	0
7	3	1	2	0
3	5	1	2	2

**Table 5:** WOA thresholds for offloading decision-making in case of an energy balancing service.

Parameter Name	Specific Threshold
Euclidean distance	<10
Round-trip time (RTT)	<90 ms
Number of epochs	<250
Population size	<20

**Table 6:** Task-offloading decision table.

Source Node ID	Target Node ID (Offloading)	Initial Distance	Initial RTT	Final Distance	Final RTT	RTT Reduction
Cloud_node_2	Fog_node_5	12.01	93.03 ms	6.07	89.71 ms	−3.32 Ms
Cloud_node_8	Fog_node_10	6.05	100.00 ms	5.02	75.08 ms	−24.92 Ms
Cloud_node_10	Fog_node_9	5.09	91.30 ms	6.14	78.03 ms	−13.27 Ms
Fog_node_8	Edge_node_17	11.01	90.05 ms	4.51	76.85 ms	−13.20 Ms
Fog_node_9	Edge_node_9	10.05	92.45 ms	5.86	73.20 ms	−19.25 Ms
Fog_node_10	Edge_node_2	11.05	93.05 ms	4.31	82.01 ms	−11.04 Ms

**Table 7:** DC distribution in terms of cloud nodes.

Layer	No. Nodes		Node Computational Resources			Data Connection with Lower Layers
	Scenario 1	Scenario 2	Processor	RAM	Storage	
Edge	50	70	30–50 GHz	2–8 GB	8–16 GB	N/A
Fog	30	35	40–50 GHz	4–16 GB	16–32 GB	Links to edge Latency: 50–100 ms Bandwidth: 1–10 Mbps Distance: 200–500 km
Cloud	20	20	45–50 GHz	8–32 GB	20–32 GB	Links to fog Latency: 70–100 ms Bandwidth: 100–1000 Mbps Distance: 500–1000 km

## Data Availability

The raw data supporting the conclusions of this article will be made available by the authors upon request.
